# Clinical efficacy of uniportal and biportal endoscopic ULBD techniques in the treatment of lumbar spinal stenosis

**DOI:** 10.1590/1414-431X2026e15561

**Published:** 2026-09-07

**Authors:** Penghui Ni, Xueguo Chen, Wenwen Huang

**Affiliations:** 1 Department of Spine Surgery, Jingmen Central Hospital/Jingmen Central Hospital Affiliated to Jingchu University of Technology, Jingmen, Hubei, China; 2 Department of Orthopedics, Jingmen Duodao People's Hospital, Jingmen, Hubei, China; 3 Department of Trauma Orthopedics, Jingmen Central Hospital/Jingmen Central Hospital Affiliated to Jingchu University of Technology, Jingmen, Hubei, China

**Keywords:** Lumbar spinal stenosis, Uniportal endoscopy, Biportal endoscopy, Unilateral laminotomy for bilateral decompression, Clinical efficacy

## Abstract

This study aimed to compare the clinical efficacy of unilateral laminotomy for bilateral decompression (ULBD) performed using unilateral endoscopy (UE) versus unilateral biportal endoscopy (UBE) in patients with lumbar spinal stenosis (LSS). Sixty LSS patients were randomly assigned to a UE-ULBD group or a UBE-ULBD group (30 cases each). Intraoperative blood loss, operative time, time to first ambulation, length of hospital stay, and intraoperative/postoperative complications were recorded. Low back and leg pain were assessed using the visual analog scale (VAS), and functional status was evaluated using the Oswestry Disability Index (ODI) prior to surgery and at 3 and 6 months postoperatively. Quality of life was measured by the Swiss Spinal Stenosis (SSS) questionnaire preoperatively and at 6 months post-surgery. Surgical outcomes were evaluated using the modified MacNab criteria. No differences were found between groups for intraoperative blood loss, operative time, time to first ambulation, length of hospital stay, or intraoperative/postoperative complication rates (P>0.05). Compared with preoperative values, both groups showed notable reductions in VAS and ODI scores at 3 and 6 months postoperatively (P<0.05). At 6 months postoperatively, all SSS dimension scores were significantly improved in both groups (P<0.05), and no intergroup differences were detected (P>0.05). The modified MacNab excellent-to-good rates were 93.33% in the UBE-ULBD group and 90% in the UE-ULBD group, with no significant between-group difference (P>0.05). UE-ULBD and UBE-ULBD represent reliable minimally invasive options for the surgical management of LSS.

## Introduction

Lumbar spinal stenosis (LSS) is a progressive degenerative disorder characterized by narrowing of the spinal canal and compression of neural elements, clinically manifested by chronic low back pain, radicular symptoms, and neurogenic intermittent claudication (1). With population aging, the prevalence of LSS continues to rise, resulting in substantial functional impairment and diminished quality of life. Although conservative treatment - including activity modification, nonsteroidal anti-inflammatory drugs, and rehabilitation - may alleviate symptoms in mild cases, its long-term effectiveness remains limited in patients with moderate-to-severe stenosis (2). Consequently, surgical decompression remains the primary treatment option for patients with refractory or progressive symptoms. Conventional open laminectomy has historically been the standard surgical procedure for lumbar canal decompression. While it can effectively enlarge the spinal canal and relieve neural compression, extensive muscle dissection and resection of posterior bony and ligamentous structures may compromise spinal stability and delay postoperative recovery (3,4). These limitations have stimulated growing interest in less invasive surgical strategies that aim to achieve adequate decompression while preserving normal spinal anatomy.

With advances in spinal endoscopic techniques, minimally invasive decompression has become increasingly feasible and widely adopted. Endoscopic approaches are associated with reduced soft tissue injury, postoperative pain, and length of hospital stay, while maintaining favorable clinical outcomes (5). Unilateral laminotomy for bilateral decompression (ULBD), which preserves midline structures and minimizes muscle trauma, has gained acceptance as an effective technique for LSS management (6). By enabling bilateral spinal canal decompression through a unilateral approach, ULBD theoretically offers superior preservation of spinal stability and faster postoperative recovery.

Currently, ULBD can be performed using either uniportal endoscopy (UE) or unilateral biportal endoscopy (UBE) (7). UE employs a single working channel that integrates visualization and instrumentation, whereas UBE utilizes two independent portals to separately accommodate the endoscope and surgical instruments (8). Both techniques have demonstrated favorable clinical and radiological outcomes in the treatment of LSS (7,8). UBE has also been increasingly recognized as a novel minimally invasive approach with potential advantages in visualization, irrigation, and surgical flexibility (9). However, direct comparative evidence between UE-ULBD and UBE-ULBD remains limited, and their relative advantages regarding operative efficiency, perioperative morbidity, and early functional recovery are not well established.

Therefore, a well-designed head-to-head comparison of UE-ULBD and UBE-ULBD is clinically warranted. Clarifying whether one technique offers superior operative efficiency, reduced tissue trauma, faster recovery, or improved functional outcomes would provide valuable guidance for surgical decision-making and technique selection. In this context, the present study aimed to compare the perioperative parameters, clinical outcomes, and quality-of-life improvements associated with UE-ULBD and UBE-ULBD in patients with LSS, and to further evaluate the effectiveness of bilateral decompression achieved by these two minimally invasive approaches.

## Material and Methods

### General information

Sixty patients with LSS who underwent endoscopic ULBD in Jingmen Central Hospital from April 2023 to April 2025 were enrolled. Patients were randomly allocated to the UE-ULBD group or the UBE-ULBD group using a random number table (30 cases each). There were no significant intergroup differences in age, body mass index, sex, involved spinal level, type of stenosis, Schizas grade, or symptom duration (P>0.05), indicating comparable baseline characteristics between the two groups (Table 1).

**Table 1:** Comparison of general information between patient groups treated with unilateral laminotomy for bilateral decompression (ULBD) using unilateral endoscopy (UE) or unilateral biportal endoscopy (UBE).

Indicator	UBE-ULBD group (n=30)	UE-ULBD group (n=30)	*t*/χ^2^	P
Age (years)	59.97±9.93	61.63±8.53	0.698	0.488
Body mass index (kg/m^2^)	25.27±3.02	24.57±2.84	0.928	0.357
Sex			*	0.606
Male	16 (53.33)	13 (43.33)	-	-
Female	14 (46.67)	17 (56.67)	-	-
Educational level			*	0.828
Primary school	8 (26.67)	10 (33.33)	-	-
Junior high school	15 (50.00)	12 (40.00)	-	-
Senior high school	6 (20.00)	6 (20.00)	-	-
College and above	1 (3.33)	2 (6.67)	-	-
Affected spinal segment			*	0.262
L3/4	0 (0.00)	2 (6.67)	-	-
L4/5	25 (83.33)	25 (83.33)	-	-
L3/4 + L4/5	2 (6.67)	0 (0.00)	-	-
L5/S1	3 (10.00)	3 (10.00)	-	-
Stenosis type			*	0.572
Central type	14 (46.67)	10 (33.33)	-	-
Lateral recess type	7 (23.33)	9 (30.00)	-	-
Mixed type	9 (30.00)	11 (36.67)	-	-
Schizas grade			*	0.592
Grade B	18 (60.00)	20 (66.67)	-	-
Grade C	12 (40.00)	10 (33.33)	-	-
Symptom duration			*	0.347
<6 months	5 (16.67)	8 (26.67)	-	-
≥6 months	25 (83.33)	22 (73.33)	-	-

Data are reported as means±SD or number and percentage. Student's *t*-test or chi-squared test. *Fisher's exact test.

This study was approved by Jingmen Central Hospital Ethics Committee, and all patients provided written informed consent.

### Inclusion criteria

Patients were eligible for inclusion if they met all of the following criteria: 1) a confirmed diagnosis of LSS according to established diagnostic criteria (10), with typical clinical manifestations including intermittent claudication, low back pain and radiating pain, and numbness or weakness of the lower extremities, supported by lumbar CT or MRI findings demonstrating central canal, lateral recess, or foraminal stenosis consistent with clinical symptoms and signs; 2) involvement of a single spinal level or two adjacent levels, with a Schizas classification of grade B or C; 3) failure of conservative treatment (including pharmacological therapy, physical therapy, and/or epidural injections) for at least 3 months, or recurrent symptoms despite conservative management; 4) age between 18 and 80 years, with preoperative assessment indicating adequate general condition to tolerate endoscopic ULBD, clear consciousness, and ability to comply with perioperative management and follow-up; 5) no prior history of lumbar spine surgery; 6) provision of written informed consent following approval by the institutional ethics committee.

### Exclusion criteria

Patients were excluded if any of the following conditions were present: 1) lumbar instability defined as vertebral translation ≥3 mm or angular motion >15° on dynamic radiographs, grade II or higher spondylolisthesis, scoliosis (Cobb angle >10°), spinal infection, tuberculosis, tumor, or fracture; 2) lower extremity pain or numbness primarily attributable to severe hip or knee joint disease; 3) local infection, ulceration, or sinus tract involving lumbar skin or soft tissues; 4) coagulation disorders, marked bleeding tendency, or long-term use of anticoagulant therapy (e.g., warfarin, rivaroxaban) that could not be safely discontinued; 5) severe dysfunction of major organs (heart, liver, kidney, or lung), or serious comorbidities such as uncontrolled diabetes, malignant hypertension, or psychiatric disorders precluding tolerance of surgery or adherence to follow-up; 6) intraspinal adhesions or congenital/anatomical abnormalities; 7) severe osteoporosis defined as a T-score ≤ −2.5 on dual-energy X-ray absorptiometry; 8) pregnancy, lactation, or inability to complete follow-up and questionnaire assessments due to cognitive impairment, psychiatric illness, or other causes; 9) incomplete clinical or imaging data.

### Methods

All surgical procedures were performed by the same senior spine surgeon with extensive experience in spinal endoscopy. General anesthesia was administered to all patients, who were placed in the standard prone position with the abdomen suspended. Under fluoroscopic guidance using a C-arm X-ray system (Cios Alpha, Siemens, Germany), the target intervertebral level and interlaminar space were precisely localized and marked on the body surface.

#### UE-ULBD group

Procedures in the UE-ULBD group were performed using a single-channel full-endoscopic system (Delta System, Joimax, Germany). The symptomatic side or the side with more severe stenosis was selected as the approach side. A longitudinal skin incision approximately 8 mm in length was made 1.0-1.5 cm lateral to the posterior midline. Following sequential dilation of the paraspinal soft tissues, a working cannula (outer diameter, 9.5 mm) was introduced, and a 30° wide-angle endoscope connected to an imaging system (IMAGE1 S, Storz, Germany) was inserted. Under endoscopic visualization, soft tissues overlying the interlaminar space were debrided using a radiofrequency ablation electrode (Vulcan EASy, ArthroCare, USA) to establish the initial working corridor. Ipsilateral decompression was performed first. The upper margin of the caudal lamina, the lower margin of the cranial lamina, and the medial portion of the inferior articular process were sequentially removed using an endoscopic high-speed burr (Legend EHS, Stryker, USA) or endoscopic rongeurs, thereby exposing and resecting the superficial layer of the ligamentum flavum. After careful identification and protection of the exiting nerve root, hypertrophic bone on the medial aspect of the superior articular process was further removed to enlarge the lateral recess until the medial wall of the ipsilateral pedicle was clearly visualized. Subsequently, contralateral decompression was performed. The ventral bone at the base of the spinous process was removed using a burr or osteotome to cross the midline. The attachment of the contralateral ligamentum flavum to the ventral lamina and the characteristic V-shaped cleft were identified as midline landmarks. The deep layer of the ligamentum flavum was carefully dissected and excised to expose the contralateral dural sac. The medial portion of the contralateral inferior articular process and the ventral hypertrophic bone of the contralateral superior articular process were then removed to complete contralateral lateral recess decompression until the medial wall of the contralateral pedicle was exposed. Adequate decompression of the bilateral spinal canal and neural foramina was achieved throughout the procedure using “coaxial” or “eccentric triangle” manipulation techniques under endoscopic visualization through a single incision. Concomitant discectomy was performed when significant disc herniation was present. After meticulous hemostasis, the endoscope and working cannula were withdrawn, the incision was closed with a single suture, and sterile dressings were applied.

#### UBE-ULBD group

Procedures in the UBE-ULBD group were performed using a dual-channel endoscopic system. The endoscopic visualization system (IMAGE1 S, Storz) was independent of the dedicated working channel for surgical instruments. The symptomatic side was selected as the primary operative side. Two longitudinal skin incisions were made approximately 0.5 and 3.0 cm lateral to the posterior midline, respectively. The incision for the visualization portal measured approximately 4-6 mm, whereas that for the working portal measured 8-10 mm in length. The longitudinal distance between the two incisions was approximately 2.5-3.0 cm. A 30° endoscope was introduced through the visualization portal. Through the working portal, a dedicated dissector was inserted, and the multifidus muscle was bluntly separated from the surfaces of the spinous process and lamina. Under fluoroscopic guidance, dilators were advanced to the target interlaminar space. After the establishment of a continuous saline irrigation working environment, soft tissues within the visual field were initially debrided using radiofrequency ablation. The sequence and principles of decompression were consistent with those used in the UE-ULBD group. Ipsilateral decompression was performed first by resecting the ipsilateral lamina, the medial portion of the facet joint, and the ligamentum flavum. Subsequently, the ventral bone at the base of the spinous process was resected to serve as a “bridge” for contralateral access. Contralateral decompression was then completed by removing the contralateral ligamentum flavum and hypertrophic facet joint components. The biportal technique enabled bimanual coordination under endoscopic visualization - typically with suction held in one hand and a high-speed burr or rongeur in the other - thereby improving efficiency in managing dense osteophytes. Adequate decompression was defined by restoration of bilateral dural sac pulsation, relaxation of the nerve roots, and visualization of the medial walls of the pedicles bilaterally. Management of intervertebral disc herniation was identical to that in the UE-ULBD group. At the completion of surgery, a drainage tube was placed through the working portal, and the fascia and skin were closed in layers.

#### Postoperative management

After surgery, all patients in both groups received routine postoperative care, including prophylactic antibiotics, dehydration therapy, and analgesia. Drainage tubes, if placed, were removed within 24-48 h postoperatively. Under medical supervision, patients were encouraged to ambulate with a lumbar brace beginning on the first postoperative day and to perform progressive lumbar extensor muscle strengthening exercises.

### Outcome measures

#### Perioperative indicators

Perioperative variables recorded and compared between the two groups included intraoperative blood loss, operative time, time to first ambulation, and postoperative length of hospital stay. Intraoperative blood loss was estimated by subtracting the volume of irrigation fluid from the total volume collected by suction, combined with the weight of blood-soaked gauze. Operative time was defined as the duration from skin incision to completion of wound closure. Time to first ambulation was defined as the interval from the end of surgery to the first independent standing or walking with assistance. Postoperative length of stay was calculated from the day of surgery (designated as Day 0) to discharge, based on criteria including satisfactory wound healing, stable vital signs, independent ambulation, and tolerable pain.

#### Complications

All intraoperative and postoperative complications were documented in detail. Intraoperative complications included dural tears, nerve root injury, rupture and bleeding of the epidural venous plexus, and cerebrospinal fluid leakage. Postoperative complications included symptomatic epidural hematoma, incision infection or delayed healing, recurrent disc herniation at the decompressed level, persistent or aggravated neurological deficits, and postoperative paresthesia. The incidence and number of complications were calculated for each group.

#### Pain assessment

Lumbar and leg pain were assessed preoperatively and at 3 and 6 months postoperatively using the visual analog scale (VAS) (11) with scores ranging from 0 (no pain) to 10 (unbearable pain).

#### Functional assessment

Lumbar function was evaluated using the Oswestry Disability Index (ODI) (12) prior to surgery and at 3 and 6 months postoperatively. The ODI consists of 10 domains, including pain intensity, personal care, lifting, walking, sitting, standing, sleeping, social life, sexual life, and traveling. Scores were converted to percentages (0-100%), with higher values indicating greater disability.

#### Quality of life

Quality of life was assessed preoperatively and at 6 months postoperatively using the Swiss Spinal Stenosis Questionnaire (SSS) (13). The SSS comprises three domains: symptom severity (items 1-7; items 1-6 scored from 1 to 5, and item 7 scored as 1, 3, or 5), physical function (items 8-12; each scored from 1 to 4), and satisfaction (items 13-18; each scored from 1 to 4). Domain scores were calculated by dividing the total domain score by the number of items, with higher scores indicating more severe symptoms, poorer functional status, or lower satisfaction.

#### Excellent-to-good rate

Overall clinical efficacy (excellent-to-good rate) at 6 months postoperatively was evaluated using the modified MacNab criteria (14), which classify outcomes into four categories: excellent, good, fair, and poor. “Excellent” was defined as complete resolution of clinical symptoms and pain with recovery of neurological function and resumption of normal daily activities. “Good” indicated resolution of symptoms and pain with return to routine activities without the need for analgesics. “Fair” referred to marked improvement in symptoms and pain but continued requirement for nonsteroidal anti-inflammatory drugs that interfered with daily activities. “Poor” indicated no improvement or worsening of symptoms requiring opioid analgesics and marked limitation of daily activities. The excellent-to-good rate was calculated as the proportion of patients classified as excellent or good among all participants.

### Statistical analysis

All data were processed using SPSS 27.0 (IBM, USA). Categorical variables were summarized as counts and proportions [n (%)], and group differences were examined using either the chi-squared test or Fisher's exact test, depending on expected frequencies. Distributional characteristics of continuous variables were analyzed with the Shapiro-Wilk test. Variables failing to meet normality assumptions are reported as medians with interquartile ranges and were compared between groups using the Mann-Whitney U test and within groups using the Wilcoxon signed-rank test. For normally distributed variables, data are reported as means with standard deviations. Variance homogeneity was evaluated prior to analysis. Where variances were homogeneous, group comparisons were performed using the independent samples *t*-test; otherwise, the Welch correction was applied. Paired observations were assessed using the paired *t*-test. Statistical significance was defined as P<0.05.

## Results

### Perioperative indicators

No differences were found between the UE-ULBD and UBE-ULBD groups in terms of intraoperative blood loss, operative time, time to first ambulation, or length of hospital stay (P>0.05; Table 2).

**Table 2:** Comparison of perioperative indicators between patient groups treated with unilateral laminotomy for bilateral decompression (ULBD) using unilateral endoscopy (UE) or unilateral biportal endoscopy (UBE).

Indicator	UBE-ULBD group (n=30)	UE-ULBD group (n=30)	*t*/Z	P
Intraoperative blood loss (mL)	65.80±16.23	68.23±17.45	0.559	0.578
Operative time (min)*	94.13±16.73	96.50±14.16	0.591	0.556
Time to first ambulation (d)	3.00 (3.00, 3.00)	3.00 (3.00, 3.00)	0.416	0.677
Length of hospital stay (d)	5.00 (4.00, 6.00)	5.00 (5.00, 6.00)	0.439	0.661

Data are reported as means±SD for normally distributed variables or as median (interquartile range) for non-normally distributed variables. Intergroup comparisons of normally distributed variables were performed using the independent samples *t*-test, while those of non-normally distributed variables were performed using the Mann-Whitney U test. *Endoscopic decompression operation time, excluding the time for preoperative positioning, disinfection, draping, and postoperative suture.

### Incidence of complications

Intraoperatively, dural tear occurred in 1 patient (3.33%) in the UE-ULBD group during removal of a calcified ligamentum flavum, resulting in the appearance of clear cerebrospinal fluid in the operative field. The patient was immediately repositioned to reduce intraspinal pressure, and the defect was sealed endoscopically using fibrin glue combined with a gelatin sponge. Postoperatively, the patient was managed conservatively with a head-down positioning protocol and increased fluid replacement. No symptomatic cerebrospinal fluid leakage or pseudomeningocele developed, and the surgical incision healed uneventfully. No intraoperative complications were observed in the UBE-ULBD group.

Postoperatively, one patient (3.33%) in the UE-ULBD group developed a superficial wound infection manifested by localized redness, swelling, and minimal exudation on postoperative day 5; the infection resolved following intensified wound care and oral antibiotic therapy. Another patient (3.33%) experienced new-onset lower limb numbness, which was attributed to transient nerve root traction or edema; symptoms completely subsided within two weeks after treatment with methylcobalamin and short-term corticosteroid therapy. One additional patient (3.33%) reported anterolateral thigh pain on the operative side, which improved with nonsteroidal anti-inflammatory medication and physical rehabilitation. In the UBE-ULBD group, one patient (3.33%) developed a superficial wound infection and was managed using the same treatment protocol. Another patient (3.33%) reported exacerbation of radiating pain in the right lower extremity; follow-up MRI excluded hematoma formation and inadequate decompression, and the symptoms were attributed to nerve root irritation, which markedly improved after one month of conservative management.

No differences were found in the incidence of intraoperative and postoperative complications between the two groups (P>0.05).

### Pain levels and lumbar function

Baseline lumbar and leg VAS scores as well as ODI values were comparable between the two groups (P>0.05). Compared to preoperative levels, both cohorts exhibited marked reductions in lumbar and leg pain and significant improvement in functional status at 3 and 6 months following surgery (P<0.05). No significant differences were noted between the UE-ULBD and UBE-ULBD groups at any postoperative time point (P>0.05) (Table 3).

**Table 3:** Comparison of pain levels and lumbar function by visual analog scale (VAS) and Oswestry Disability Index (ODI) between patient groups treated with unilateral laminotomy for bilateral decompression (ULBD) using unilateral endoscopy (UE) or unilateral biportal endoscopy (UBE).

Time	UBE-ULBD group (n=30)	UE-ULBD group (n=30)	Z	P
Preoperatively				
Lumbar VAS (score)	7.00 (6.00, 7.00)	7.00 (6.00, 7.00)	0.924	0.356
Leg VAS (score)	7.00 (6.00, 8.00)	7.00 (6.00, 8.00)	0.386	0.700
ODI (%)	65.00 (60.00, 69.00)	63.50 (58.00, 68.00)	1.267	0.205
At 3 months postoperatively				
Lumbar VAS (score)	2.00 (2.00, 3.00)^a^	2.00 (2.00, 3.00)^a^	0.326	0.745
Leg VAS (score)	2.00 (1.00, 2.00)^a^	2.00 (1.00, 2.00)^a^	0.935	0.350
ODI (%)	20.00 (18.00, 23.00)^a^	22.00 (19.00, 24.00)^a^	1.590	0.112
At 6 months postoperatively				
Lumbar VAS (score)	1.00 (1.00, 2.00)^a^	1.00 (1.00, 2.00)^a^	0.800	0.424
Leg VAS (score)	1.00 (0.00, 1.00)^a^	1.00 (0.00, 1.00)^a^	0.257	0.797
ODI (%)	14.50 (12.00, 16.00)^a^	15.50 (13.00, 17.00)^a^	1.671	0.095

Non-normally distributed variables are reported as median (interquartile range). Pairwise comparisons were conducted using the Mann-Whitney U test (between groups) or the Wilcoxon signed-rank test (within groups). ^a^P<0.05 *vs* the preoperative values in the same group.

### Quality of life

Preoperative SSS domain scores did not differ significantly between the two groups (P>0.05). At 6 months postoperatively, all SSS domain scores were significantly improved in both groups compared with baseline values (P<0.05). Intergroup comparisons revealed no statistically significant differences in any SSS domain at follow-up (P>0.05) (Table 4).

**Table 4:** Comparison of quality of life scores between patient groups treated with unilateral laminotomy for bilateral decompression (ULBD) using unilateral endoscopy (UE) or unilateral biportal endoscopy (UBE).

Time	UBE-ULBD group (n=30)	UE-ULBD group (n=30)	*t*	P
Preoperatively				
Symptom severity	3.23±0.25	3.29±0.25	0.926	0.358
Physical function	2.90±0.25	3.02±0.25	1.908	0.061
Satisfaction	3.12±0.22	3.21±0.24	1.547	0.127
At 6 months postoperatively				
Symptom severity	1.62±0.30^a^	1.70±0.31^a^	1.056	0.295
Physical function	1.54±0.30^a^	1.63±0.31^a^	1.111	0.271
Satisfaction	1.62±0.34^a^	1.72±0.36^a^	1.036	0.304

Normally distributed variables are reported as means±SD. Intergroup comparisons were performed using the independent samples *t*-test, and within-group comparisons were performed using the paired samples *t*-test. ^a^P<0.05 *vs* the preoperative values in the same group.

### Excellent-to-good rates

According to the modified MacNab criteria, the excellent-to-good rate at 6 months was 93.33% in the UBE-ULBD group and 90.00% in the UE-ULBD group, with no statistically significant difference between groups (P>0.05; Table 5).

**Table 5:** Comparison of excellent-to-good rates [n (%)] between patient groups treated with unilateral laminotomy for bilateral decompression (ULBD) using unilateral endoscopy (UE) or unilateral biportal endoscopy (UBE).

Grade	UBE-ULBD group (n=30)	UE-ULBD group (n=30)	P
Excellent	18 (60.00)	16 (53.33)	-
Good	10 (33.33)	11 (36.67)	-
Fair	2 (6.67)	3 (10.00)	-
Poor	0 (0.00)	0 (0.00)	-
Excellent and good rate	28 (93.33)	27 (90.00)	1.000

Data are reported as n (%). Between the two groups, only the excellent-plus-good rate was compared statistically using Fisher's exact test. The symbol “-” in the table indicates that no separate statistical test was performed for individual efficacy grades.

## Discussion

Our findings demonstrated that both UE-ULBD and UBE-ULBD provided comparable perioperative performance and early postoperative recovery in patients with LSS. No significant differences were observed between the two techniques in terms of intraoperative blood loss, operative duration, time to first ambulation, or postoperative length of hospital stay, implying that surgical approach selection may be guided by surgeon's experience, equipment availability, and institutional preferences rather than expectations of perioperative superiority.

Microscopic and endoscopic ULBD techniques have been shown to mitigate iatrogenic spinal instability and yield more favorable clinical outcomes compared with conventional open laminectomy (15). Consistent with these reports, our results confirmed that endoscopic ULBD, regardless of portal configuration, achieved effective bilateral decompression with high procedural safety. Although navigation-assisted UBE-ULBD has been reported to enhance surgical efficiency and reduce radiation exposure without compromising outcomes (16), our findings indicated that comparable perioperative profiles can also be achieved using UE-ULBD when performed by experienced surgeons. This suggests that the clinical benefits of ULBD are primarily attributable to the decompression strategy itself rather than the specific endoscopic portal configuration.

The safety profiles observed in our cohort further support the reliability of both techniques. Previous large-scale studies have reported overall complication rates of approximately 8.1% for UBE, with dural tears being the most frequent complication (17), while transient neurological deficits and symptomatic epidural hematomas occur at relatively low frequencies. In our study, complication rates were low and comparable between the two groups, and all major complications were managed conservatively with favorable prognoses. These findings emphasized that, in experienced hands, both UE-ULBD and UBE-ULBD can be performed with high procedural safety and predictable recovery.

Pain relief and functional restoration are primary goals of surgical intervention in LSS. A prospective comparison of bilateral laminotomy, unilateral laminotomy, and laminectomy demonstrated that laminotomy-based techniques are associated with lower complication rates and reduced residual pain (18). In alignment with these observations, our results showed that both UE-ULBD and UBE-ULBD significantly alleviated lumbar and leg pain and improved ODI scores, with no detectable difference in therapeutic efficacy between the two groups. These findings support the clinical equivalence of the two minimally invasive strategies in achieving meaningful symptomatic and functional improvement.

Quality-of-life outcomes further reinforce the clinical value of both approaches. Endoscopic uniportal interlaminar decompression has been associated with high patient satisfaction and a low incidence of complications (19). Additionally, the biportal endoscopic technique has gained popularity due to its independent visualization and working portals, which enhance surgical flexibility and decompression efficiency (20,21). Our disease-specific SSS assessments demonstrated significant postoperative improvements in symptom severity, physical function, and patient satisfaction in both groups, confirming that both techniques effectively address the core impairments and quality-of-life limitations associated with LSS.

From a clinical decision-making perspective, our findings provide practical guidance. Because no significant differences were identified in perioperative efficiency, safety, pain relief, functional recovery, or quality-of-life improvement, UE-ULBD and UBE-ULBD may be considered functionally equivalent options. For institutions with limited resources or constrained operating room infrastructure, UE-ULBD may offer a more economical and technically streamlined alternative. Conversely, UBE-ULBD may be favored in centers prioritizing bimanual flexibility, enhanced visualization, and surgeon ergonomics. Thus, individualized selection based on institutional capacity and surgeon expertise appears to be a rational approach.

Several limitations should be acknowledged. The sample size of 60 patients may limit the detection of rare adverse events or subtle intergroup differences. Additionally, economic analyses were not performed, precluding cost-effectiveness comparisons that could further inform institutional decision-making and resource allocation. Future multicenter studies with larger cohorts, extended follow-up, and formal health economic evaluations are warranted to refine surgical selection strategies.

In summary, both UE-ULBD and UBE-ULBD provide safe, efficient, and clinically effective bilateral decompression for lumbar spinal stenosis, yielding significant improvements in pain, function, and quality of life. These two minimally invasive approaches should be regarded as complementary rather than competitive techniques, offering flexible and reliable options for the surgical management of LSS.

## Data Availability

The datasets generated and/or analyzed during the current study are available from the corresponding author on reasonable request.
